# Biological Applications of Severely Plastically Deformed Nano-Grained Medical Devices: A Review

**DOI:** 10.3390/nano11030748

**Published:** 2021-03-16

**Authors:** Katayoon Kalantari, Bahram Saleh, Thomas J. Webster

**Affiliations:** 1 Department of Chemical Engineering, Northeastern University, Boston, MA 02115, USA; th.webster@neu.edu; 2 R&D Department, Rosies Base, Inc., Cambridge, MA 02142, USA

**Keywords:** severe plastic deformation, titanium, stainless steel, ultra-fine-grained microstructure, implants

## Abstract

Metallic materials are widely used for fabricating medical implants due to their high specific strength, biocompatibility, good corrosion properties, and fatigue resistance. Recently, titanium (Ti) and its alloys, as well as stainless steel (SS), have attracted attention from researchers because of their biocompatibility properties within the human body; however, improvements in mechanical properties while keeping other beneficial properties unchanged are still required. Severe plastic deformation (SPD) is a unique process for fabricating an ultra-fine-grained (UFG) metal with micrometer- to nanometer-level grain structures. SPD methods can substantially refine grain size and represent a promising strategy for improving biological functionality and mechanical properties. This present review paper provides an overview of different SPD techniques developed to create nano-/ultra-fine-grain-structured Ti and stainless steel for improved biomedical implant applications. Furthermore, studies will be covered that have used SPD techniques to improve bone cell proliferation and function while decreasing bacterial colonization when cultured on such nano-grained metals (without resorting to antibiotic use).

## 1. Introduction

Over the existence of mankind, enormous research has been completed to develop structural materials with extraordinary properties. These kinds of materials have been used in several areas, such as the medical, oil, civil, and aerospace fields [1]. In comparison with polymers and ceramics, metals are excellent candidates as load-bearing implants for improving the biomedical engineering field [2]. Nowadays, metallic biomaterials, including stainless steel (SS), titanium, and its alloys, have been widely applied from tooth fillings and root implants in dental applications to vascular stents, as well as in orthopedic surgery as total joint replacements, bone screws, plates, and pins. Metallic implants satisfy numerous specific criteria for clinical use, including wear resistance and high corrosion, biocompatibility, appropriate ductility, and strength properties. However, while numerous studies have been completed on implantable metals, only a few metals can be used [3,4]. Although most metallic or ceramic implants (such as zirconium oxide and aluminum oxide) do not corrode in the air, their high rate of corrosion and metallic ion release limits their use in the body. By establishing a strong bond between local tissue and implants (which corrosion would inhibit), a higher rate of healing and lower implant rejection can be reached. These implants are a potential class of materials for the regeneration of hard tissues and are known as bioactive [4]. Currently, titanium alloys, hydroxylapatite, calcium phosphate, and bioactive glasses are all well-known bioactive implantable materials.

To solve the aforementioned limitations, bioactive surface coatings have been adopted as a strategy, but delamination and crack formation from negative thermal effects while coating metals have been crucial parameters to consider [5]. Furthermore, the fabrication of the metallic implant itself to be more bioactive is another feasible approach, and is more attractive for developing bioactive metallic implants. Another type of biomaterial is termed biotolerant (such as CrCo alloys, stainless steel, and polymethyl-methacrylate), and it releases elements in a nontoxic concentration, but may result in fibrous capsule formation, thus also inhibiting new bone growth [6]. 

Along the lines of fabricating the metallic implant itself to be more bioactive, changing metal crystalline grain size has also been shown to alter biological properties [7,8,9,10,11]. In fact, surfaces with ultra-fine-grained (UFG) materials are known to possess altered surface energy and modify initial protein adsorption to control or inhibit the adhesion and long-term functioning of cells. The incremental increases in surface area that result from nanostructured materials in addition to greater surface reactivity are inherent aspects of UFG materials for promoting their interactions with cells [12]. Gleiter [13] was one of the first to state that, with a reduction in material grain size to the nanoscale, significant changes in the physical and mechanical properties of materials could be achieved. Nanostructured materials typically show significant strength, but their ductility is usually not high. Recently, several studies have reported improvements in ductility without sacrificing nanograined material strength [13,14]. When grain size is reduced, the fraction of grain boundaries at the surface will increase drastically. During the process of nanostructuring, more lattice imperfections, including dislocations, vacancies, twins, and stacking faults, are formed, along with a reduction in grain size. Thus, changes in the material properties of nanograined metals can all be related to the increased presence of the aforementioned defects. 

Another key property of a metal that is significantly changed by nanostructuring is surface energy [15]. In comparison with conventional materials, the amount of protein adsorption and subsequent cell attachment, cell proliferation, and cell differentiation is higher on materials with nanostructures [16]. So, biomaterials with nano- and/or sub-micrometer structures are known to be more beneficial for tissue growth compared with conventional ones. Moreover, the nano- and microstructures play a significant role in metallic degradation behavior, especially in metals that chemically degrade in a biological environment (such as Mg). 

**Figure 1:**
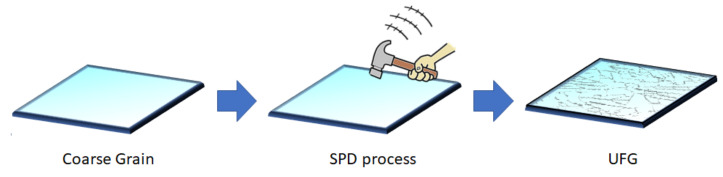
The severe plastic deformation (SPD) process can be thought of as the impact of a hammer on a window glass.

Several methods can be used to produce metals with nanostructures. All of the processing methods can be considered as top-down or bottom-up approaches. Severe plastic deformation (SPD) can be used to gradually refine grain sizes from a coarse-grained material, while in nano- or UFG materials, they can also be fabricated through the agglomeration of structural blocks of nanoparticles, atoms, or molecules. UFG materials are known as materials with grain sizes smaller than 1000 nm, and they are mostly referred to as nanostructured materials when they are built from grains smaller than one hundred nanometers [1,17]. Several of these techniques have been designed to modify implant surfaces—not just the bulk of the material—to enhance their properties [18,19]. SPD techniques are commonly applied to the bulk of the material through mechanical force, and there is no need for chemical reactions [20]. In these SPD processes, a large amount of hydrostatic pressure is applied, which leads to fabricating UFG metals with high-angle grain boundaries [21]. In 1952, Bridgman [22] reported a new technique for processing materials through the combination of shear deformation with high hydrostatic pressure, which is known as a core SPD method. Over the last twenty years, various SPD processes have been created for the refinement of metallic grains to less than a micrometer, or even in the nano-sized range [17]. The differences between such SPD processes are mostly related to the deformation behavior of the specific alloy/metal, the required processing load, and the strain imposed per pass [23]. The SPD process can be described in simple terms by considering an analogue of the impact of a hammer on a glass window (Figure 1). The material microstructure can be simulated by the window glass, and the frame of the window can be associated with the role of the hydrostatic pressure used in the SPD technique. The glass is crushed due to the impact of the hammer, which is the same as the high strain applied to the material. The crushed glass can be thought of as the refinement in the microstructure from coarse grain materials to UFG and nanostructured grain materials [24].

Therefore, to obtain a material with a very fine crystalline structure, SPD can be used as an effective approach for various crystalline materials. SPD causes the creation of micrometer- and sub-micrometer-sized grains out of the original coarse grains of the material [25]. Generally, the aspects of the SPD technique are stated as follows:

- Greater strains are imposed on the material;
- More hydrostatic pressure is applied;
- Material free flow is prevented;
- The dimensions of the material are not changed significantly during or after the process;
- Materials with micro- and/or nanostructured and high-angle grain boundaries are produced;
- Micro-structured and/or nanostructured homogeneous materials with uniform properties are fabricated;
- There are no pores, mechanical defects, or cracks in the final material [24].

Grain size has a profound influence on the mechanical properties of a material, and particularly on its strength. Micro- and nanostructure grain refinement allows one to enhance material strength without changing chemistry, which is possibly an important consideration for fast Food and Drug Administration (FDA) 510(k) approval [26]. Generally, grain size refinement leads to improved corrosion resistance, but this improvement is dependent on the processing technique applied to fabricate the refined structure [27]. It has been proven that passive films formed on UFG and/or nanostructured SS have better barrier properties. 

Recrystallization temperature can be reduced by using a cryo-rolling method favoring the formation of smaller grains while, at the same time, improving the mechanical behavior [28]. Zheng et al. [29] reported that nanocrystalline 304 SS fabricated by the equal-channel angular press (ECAP) method possessed higher corrosion resistance in a H_2_SO_4_ solution (0.5 M). This improvement in passive oxide barrier properties was related to its more compact nature and stability. Similarly, Pisarek et al. [30] showed that SS (316 L) synthesized by hydrostatic extrusion possessed a region with extended stable passivity (~0.4 V) in a +0.1 M NaCl borate buffer. It is now well established that UFG materials processed by ECAP possess improved strength and good ductility [31,32]. Hence, SPD techniques are promising for obtaining unique nano-grained materials with enhanced mechanical behavior for improving biomedical applications.

## 2. Main SPD Processes

UFG and nanostructured metals processed by SPD methods have remarkable properties [33]. Different SPD processes that have attracted great attention in the fabrication of UFG/nanostructured materials include high-pressure torsion (HPT), equal-channel angular pressing (ECAP), groove pressing (GP), ball milling (BM), accumulative roll bonding (ARB), and multi-axial forging. These processes are able to produce products of SPD in different forms, such as wires, rods, and strips, which demonstrate adaptation to existing manufacturing environments. To reach a combination of high ductility and strength and uniform plasticity in UFG metals (such as deformation at low temperatures) to suppress dynamic recovery, one must take advantage of the elevated strain rate sensitivity in the materials that are processed. Among several strategies, gradient structures in the form of gradual micro- and nano-structural elements change (such as grain size, twin thickness, and density of dislocations) and have been shown to provide great synergy in ductility–strength improvement. Numerous microstructural mechanisms have been associated with the strengthening of materials to which SPD has been applied, including the strengthening of dislocations, grain boundary and size strengthening, precipitation, second-phase dissolution, and shearable and non-shearable dispersoid particles [34,35]. A detailed explanation of each of these techniques is available in the next sections.

### 2.1. Equal-Channel Angular Press (ECAP) Process

**Figure 2:**
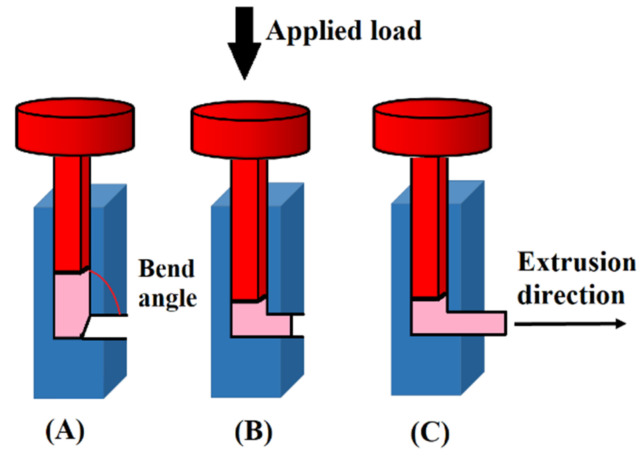
A schematic showing the working details of the equal-channel angular press (ECAP) process; first (**A**), intermediate (**B**), and final (**C**) steps.

The ECAP process was introduced in 1972 and is known as a pressing technique where metallic billets undergo plastic deformation without a reduction in their cross-section. During the ECAP process, metallic materials are pressed into a die via a continuous but kinked channel. The billet to be pressed is inserted in a die while a punch is placed on the billet top (Figure 2A) [36]. The load is used on the punch to press the billet via a channel (Figure 2B) until the billet is extruded out of the die (Figure 2C). Based on the application, the channel and billet cross-section can be rectangular or circular in shape. The extruded materials have an unaltered cross-section before and after processing, which is an interesting characteristic of ECAP, especially when a large amount of work hardening is required. While the material passes through the channel-bending portion, plastic deformation occurs. The intensity of the degree of the plastic deformation is dependent on the bend angle. The plastic strain generated from the lower bend angle is greater than that of the higher bend angle [36].

**Figure 3:**
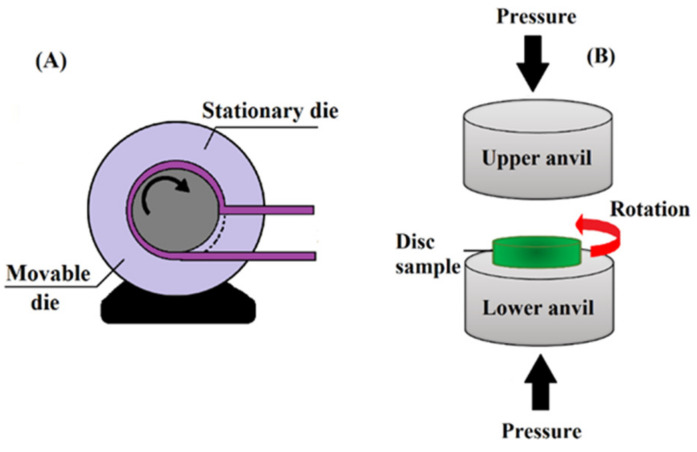
Principle of the ECAP–conformation technique (**A**); reproduced from [45]; Schematic illustration of the high-pressure torsion (HPT) process (**B**); reproduced from [46].

Presently, ECAP is the most advanced SPD method and is the only technique utilized for industrial applications. However, there are some drawbacks to ECAP. This process happens discontinuously with limitations in scale-up potential. Additionally, the portion of the product with a desired uniform structure and without cracks is rather small [37]. Thus, some processes have been proposed to improve the efficiency of ECAP, including continuous ECAP–conformation and tubular-channel angular pressing [38,39,40]. The ECAP technique allows for the production of long rods and dramatically reduces material waste during processing [41]. In this method (Figure 3A), the ECAP principle is used for a continuous cylindrical rolling process. This cylinder is located between guides into which a rod is inserted, undergoing the ECAP technique on its way out. This method is suitable for rod-shaped production at the industrial scale and is well suited for dental implants [6].

### 2.2. High-Pressure Torsion (HPT) Process

In 1937, the high-pressure torsion (HPT) technique was introduced by Bridgman [42]. In HPT (Figure 3B), while extremely high pressure is applied, the material is pressed between two anvils. Simultaneously, through the rotation of one of the anvils, shear stress is induced, while the other anvil is static, or both anvils are counter-rotated [41]. This technique is used especially for brittle and strong materials at low temperatures and under high hydrostatic pressure due to its unlimited one-step shear, which leads to the fabrication of premium micro- and nanostructures, as achieved through SPD [33]. Nevertheless, HPT has some significant disadvantages. This technique allows only for the processing of thin and small disc-shaped samples (typically, 0.2–2 in thickness and 10–20 mm in diameter), and moreover, it is a discontinuous method. A few techniques have been suggested to overcome these limitations, including continuous HPT (CHPT) for sheets or ring-shaped samples [43,44].

Another disadvantage is that it produces a significant inhomogeneous microstructure across the disc’s radius unless a large amount of strain is applied to the disc. Moreover, the processing speed is very slow, and scaling up is hard, as it involves high energy demands, pressure, and torque. However, the HPT technique has been known as an effective and desirable tool in the materials science field for a long time.

### 2.3. Hydrostatic Extrusion (HE)

**Figure 4:**
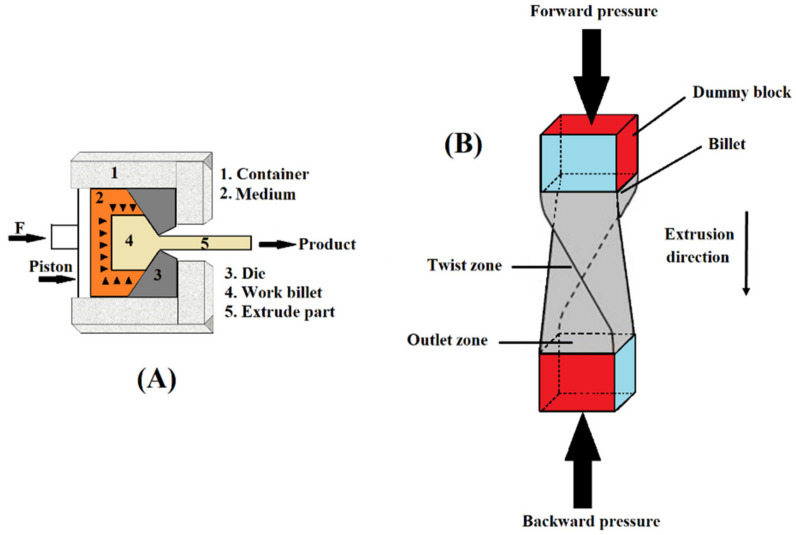
Schematic of a hydrostatic extrusion (HE) device (**A**); reproduced from [47] with permission from Elsevier, 2020. Schematic of the twist extrusion (TE) process (**B**).

The hydrostatic extrusion (HE) method has been used for almost one hundred years in industry [47]. In 1893, the HE method was patented by James Robertson, but in the 1950s, the first experiments were reported by Percy W. Bridgman [48]. This process is a direct extrusion technique, and because of the significant effect from a liquid medium, it is known as “hydraulic direct extrusion”. Simultaneously, the operational chamber is filled with a medium (usually oil) that surrounds the billet. The process of extrusion starts with the movement of pistons and the direct contact of the billet–die seals the system. With piston movement, the medium is compressed slowly, which leads to an incremental increase in the chamber’s hydrostatic pressure. The process of plastic deformation begins in the billet when the stress exceeds the yield strength of the material and the resistance from friction is overcome. Then, the billet, usually in a conical shape, is extruded via the hole of the die. The presence of the medium eliminates the direct contact between the walls of the chamber and piston with the billet; thus, frictional forces are significantly reduced [49]. Additionally, this method allows for more bulk material processing (the product and billet) than other techniques, such as HPT and ECAP. Compared to the other SPD methods, the HE technique (Figure 4A) has limitations, such as decreasing the product’s diameter. Thus, during this process, extremely high plastic deformation cannot be obtained. Other HE limitations include the loss of energy for liquid medium compaction, large requirements for a sealed structure, the necessity for fluid injection and fluid removal during every cycle of extrusion, and some further complications when extrusion is performed at higher temperatures [47].

### 2.4. Twist Extrusion (TE)

Recently, TE has attracted considerable attention. The interest in TE is attributed to a peculiar plastic flow of material that can be used for microstructural fabrication at various scales [50]. In this technique (Figure 4B), there is a die that includes a so-called twist zone located between the outlet channels and straight inlet channels, through which a billet is pressed. The twist zone’s surface is shaped by the die profile “swept” along a helix line, which leads to a “twist angle” between the outlet and inlet zones [41]. Using such a structure allows one to keep the workpiece in its original shape. While a workpiece is pressed via a TE die, plastic deformation happens on the metal through simple shear at the transient between the straight and twist channels [51]. TE works under high pressure (hydrostatic) at the deformation center. This pressure is made by using backpressure on the sample when it exits the die. This method has particularly great potential for metal grain refinement and homogenization on the microscale because of the specific deformed state of the specimens during extrusion [52].

### 2.5. Friction Stir Processing (FSP)

**Figure 5:**
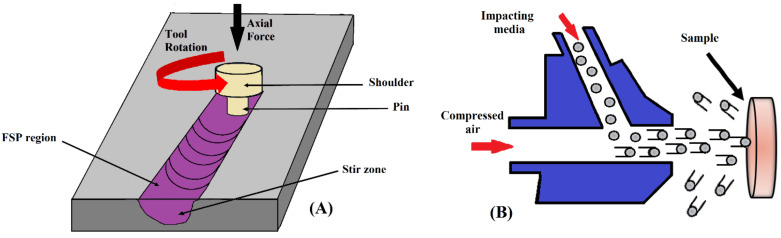
Schematic of the friction stir process (**A**) and severe shot peening (**B**); reproduced from [54], from Elsevier, 2020.

The FSP method is an efficient solid-state processing technique that provides surface layer modifications to biomedical materials. As shown in Figure 5A, this process involves penetration of materials by a rotating non-consumable tool that heats them. This localized heat leads to softening of the materials around the pin. The rotational combination of the tool gives rise to movement of the material from the pin’s front to its back. The produced heat is increased due to the friction between the workpiece and pin. In this technique, the processed zone depth can be controlled through modification of the pin tool’s length [46,53].

### 2.6. Severe Shot Peening (SSP)

The severe shot peening (SSP) technique uses a shot medium pushed at high speed onto a material’s surface [3]. In the SSP process, factors such as the dimension and the specific material of the shot, speed of the shot, the shot’s impact direction, and the shot’s coverage are specified based on preferred surface properties and the type of material to be peened. This technique is a type of conventional shot peening that uses the shot medium’s kinetic energy, as well as an increased duration of shot medium exposure, pressure, and coverage, to obtain a desirable surface [55]. SSP (Figure 5B) is known as a suitable method that can be used regardless of complex and intrinsic material shapes. The new surface layer on the materials possesses special properties in terms of strain hardening and roughness related to the compressive residual stress created upon impaction of the shot medium with the surface [56].

### 2.7. Ultrasonic Shot Peening (USSP)

**Figure 6:**
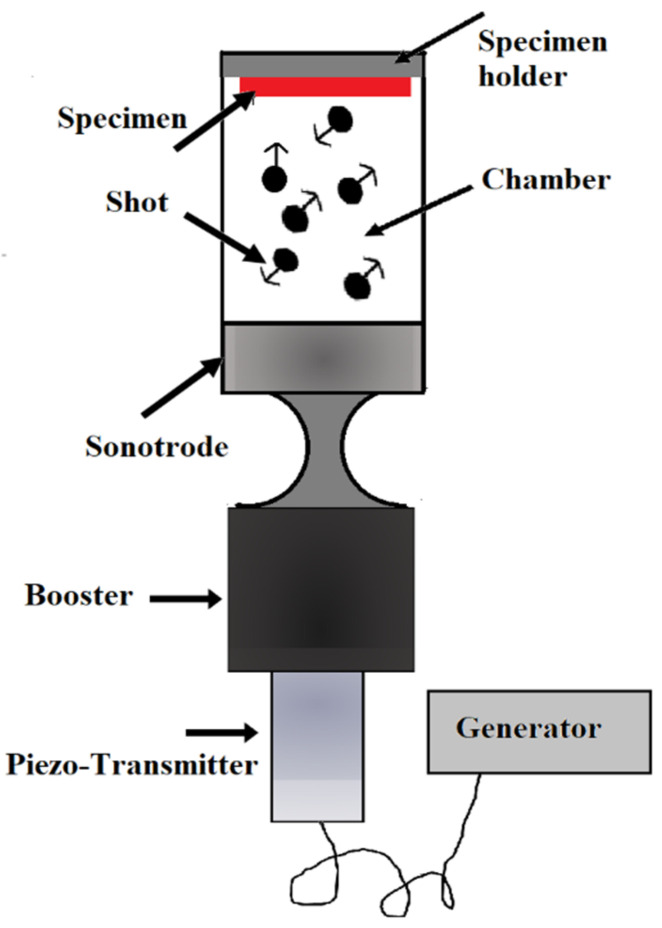
Overall view of the ultrasonic shot peening (USSP) process; reproduced from [57], with permission from Elsevier, 2020.

Ultrasonic instruments can be used to create a preferred kinetic energy of the shots to hit the surface. In this method, which is sometimes known as “surface mechanical attrition treatment” (SMAT), an ultrasonic transducer produces shots through the vibration of an ultrasonic transducer. Figure 6 shows an experimental USSP set-up.

In a reflecting chamber, there are several spherical steel balls (one-tenth of a millimeter in diameter) used with a smooth surface area. A vibration generator creates a vibrating chamber. The chamber can produce vibrations with a frequency between 50 Hz and 20 kHz. With the resonance of the balls, the surface of the sample is treated due to the flying of many balls over a short time period. The directional effects of the balls on the surface of the sample are somewhat random because the balls have random flying directions inside the chamber [56]. The main advantage of this technique includes the creation of low surface roughness because the shots are of better quality and have a lower impact speed. Moreover, the balls can be recovered after the material’s treatment and used again [57].

### 2.8. Warm Continuous Multidirectional Rolling

**Figure 7:**
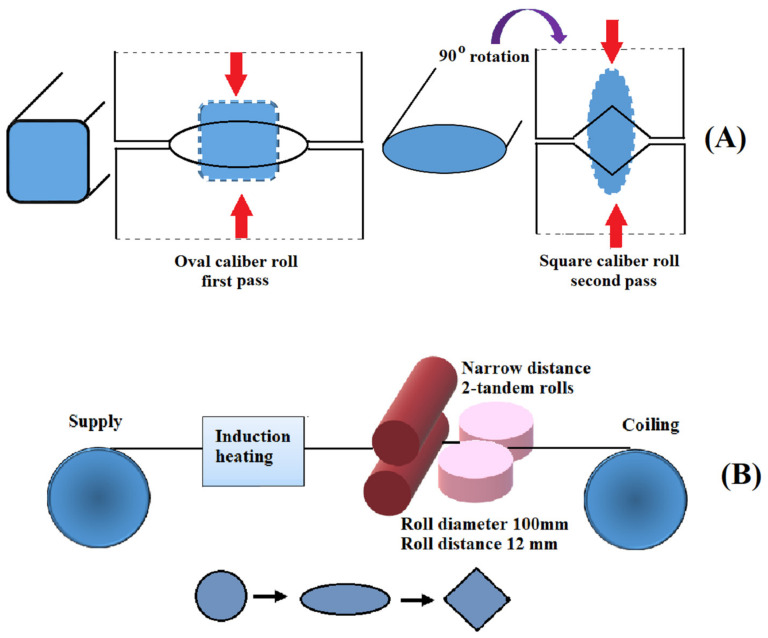
Schematic drawing of multidirectional deformation rolling realized using an oval-caliber and square-caliber rolling (**A**); redrawn from [60]. A schematic illustration of a novel advanced warm continuous oval- to square-rolling set-up (**B**); reproduced from [60], with permission from Elsevier, 2020.

The warm caliber-rolling technique is a rolling process (continuous) utilized in the production of a nanostructured wire or bar. So far, this technique has been largely utilized for the refinement of the microstructures of raw materials [58]. In 2014, Krállics et al. [59] showed that Ti created with warm caliber rolling possessed a UFG microstructure with good ductility and high tensile strength. Nevertheless, this method is not suitable for industrial quantities; thus, a more efficient and novel continuous technique with improved caliber rolling is required. By adding multidirectional compressive strain, despite the smaller reduction in thickness, more strain can be introduced into materials. To obtain long continuous products, deformation using multidirectional compressive strength is not appropriate; thus, the development of a rolling process with multidirectional deformation is required. As presented in Figure 7A, first, the flattening of a bar material occurs by rolling through a groove with an oval shape. Deformation of the materials in the opposite direction by using a square-shaped groove is the next step.

Therefore, the rolling process is equivalent to multidirectional deformation. It has been proved that in comparison with square–square grooves, this process introduces far more strain into materials [60]. To continuously fabricate a long wire via warm rolling, a prototype rolling system was designed based on this idea. Figure 7B provides a schematic drawing of this system with the following description:

- Continuous rolling (coil to coil).
- Rolling in a controlled temperature via on-line heating.
- Two-tandem rolling in the vertical and horizontal directions.
- Rolling in multiple directions with square and oval grooves.

### 2.9. Warm Multi-Pass Caliber Rolling

**Figure 8:**
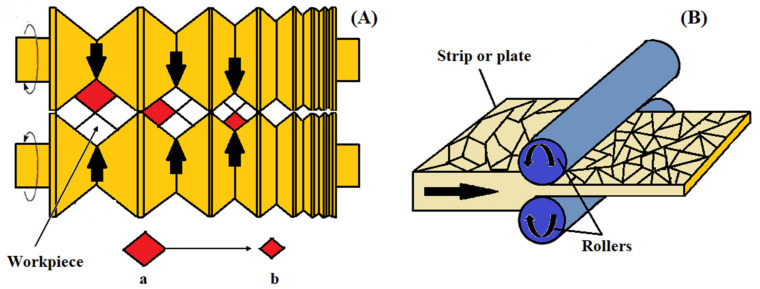
Schematic drawing of caliber rolling (**A**); reproduced from [60], with permission from Elsevier, 2020. A schematic diagram of the cold-rolling system (**B**).

For the creation of bulk UFG rods in industry, multi-pass caliber rolling has been used as an alternative to traditional SPD processes [61]. For example, Torizuka et al. [60] developed a warm multi-pass caliber-rolling process to create a nanostructured steel bar. Figure 8A provides a schematic diagram of caliber rolling, which is a simple technology that imposes a large strain using two-dimensional reduction. If a material with a section of (a × a) mm^2^ is deformed to (b × b) mm^2^, the imposed strain can be calculated with Equation (1):

ɛ = ln (a^2^/b^2^) (1)

Obviously, a large strain can be simply introduced into a material using caliber rolling.

### 2.10. Cold-Rolling Process

Cold rolling (Figure 8B) is an efficient method for deforming different metals into foil- and sheet-shaped products with improved strength and mechanical properties. In this process, it is required to use a temperature below the material’s recrystallization temperature [62,63]. The roll is the main section of the rolling procedure and generally consists of gray and alloyed iron, as well as forged and cast steel. Steel rollers are tougher and stronger than iron ones. During this process, wear, thermal and bending stress, and frictional forces are applied to the rolls. Several surface roughness values, including higher peaks (surface asperities) and lower valleys, appear on the surface of metals after machining [64]. In the cold-rolling technique, the external surface of one metallic cylinder is rolled over another; thus, the asperities of the surfaces contact each other. As a result, friction between the two metal contact surfaces occurs [65]. In this process, different factors have a non-linear effect on the single-stand rolling mill, such as the tension in the front and back, thickness, friction coefficient, and average yield stress. Any modifications of either of them will lead to a change in the rolling load and, thus, in the thickness of the product [66].

Nowadays, in industry, fine roll finishing is in high demand because of unwanted defects related to the rolls that form due to higher surface roughness. In this process, on the external surface, mechanical wear occurs due to some parameters, such as motion type and shape, contact area, and surface finish [67].

### 2.11. Accumulative Roll-Bonding Process (ARB)

**Figure 9:**
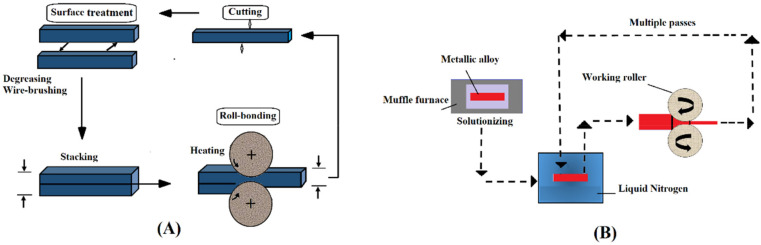
Accumulative roll bonding (**A**); reproduced from [6], with permission from Elsevier, 2020. A schematic of the cryo-rolling process (**B**); reproduced from [73], with permission from Elsevier, 2021.

A variety of deformation methods based on rolling have been introduced for grain refinement down to the ultra-fine scale. One of them is termed the accumulative roll-bonding (ARB) process. This method uses a conventional rolling instrument [46]. Figure 9A shows this process. In this technique, two metallic strips (which are similar or dissimilar to each other) are placed together and repeatedly rolled to a one-half reduction in thickness. The sheet in the rolled shape is split in two, degreased, and wire-brushed before being stacked again. The whole process of rolling should be at a high temperature with no recrystallization to make sure that the appropriate accumulative strain is achieved. The final product processed by ARB has multilayer structures that are created gradually with the ARB process [68]. Thus, ARB is known as a solid-state technique that can be used for the fabrication of particle-reinforced composites of a metal matrix with enhanced mechanical properties [69]. Moreover, the ARB process involves bonding (roll bonding) along with deformation. Nevertheless, ARB has a few limitations compared with other SPD techniques: First, this process needs a high level of technological precision and it is time consuming, and second, the bonding quality between layers affects the mechanical properties of the final sheets [41]. 

### 2.12. Cryo-Rolling (CR)

Cryo-rolling is a method that uses liquid nitrogen to create temperatures (ultra-cold temperatures) for modifying a material’s microstructure (Figure 9B). During this process, the dislocation density is increased because of the suppression of dynamic recovery. Improvements in the cryo-rolled alloy strength are related to grain refinement, higher dislocation density, and dynamic recovery suppression [70]. Among SPD processes, cryo-rolling is a unique technique that is appropriate for producing thin sheets with a UFG structure on a large and continuous scale [71]. Due to sheet rolling at liquid nitrogen temperatures, a 70–90% reduction in thickness occurs in order to obtain a high level of grain refinement. In comparison with other conventional rolling techniques conducted at room temperature, this process leads to an incremental increase in hardness and strength [72]. However, a considerable drop in formability and ductility is one of the main limitations of this process, so the produced sheets are not good candidates for industry despite their improved strength [71].

### 2.13. Constrained Groove Pressing (CGP) 

Constrained groove pressing (CGP) is a method that uses grooves at room, cryogenic, or elevated temperatures to control the microstructure and improve the mechanical properties of sheet-type UFG metals (e.g., magnesium, aluminum, copper, nickel, etc.) without changing their dimensions [74,75]. CPG is a comparatively new SDP process developed by Shin et al. [76] that subjects the specimen to repetitive shear deformation at different strains using a combination of grooved and flat dies. In this method, the distance between the upper and lower die can be the same as the sample thickness, and thus, the SPD process takes place without altering the dimensions of the sheet. It has been reported that CPG usually increases the strength, hardness, and fatigue of metals and sometimes decreases their ductility by decreasing the grain size [77,78].

**Figure 10:**
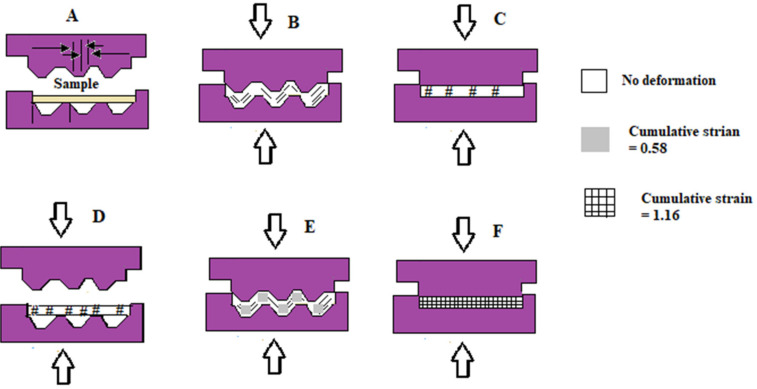
A schematic illustration of the sequences of the constrained groove-pressing (CGP) technique; groove pressing stage (**A**,**B**), sample is rotated by 180° (**D**), the successive pressings with a grooved die (**E**), a flat die (**F**), reproduced from [76,79], with permission from Elsevier, 2021.

A schematic illustration of a constrained groove-pressing technique is presented in Figure 10A–F. The sample is subjected to a simple shear deformation in the 45° diagonal surfaces between the flat surfaces and has a theoretical effective strain of 0.58. The materials along the flat surfaces were not subjected to any straining [79]. The cumulative strain in the deformed region following the second pressing becomes 1.16 [76].

## 3. Metallic Biomaterials for Medical Implants

For numerous years, metallic biomaterials have been widely used in surgery because of their excellent formability, fracture resistance, and great strength. Most metals are reactive in the human body and corrode in physiological conditions. Thus, at present, the metals utilized as implants are limited to four basic categories: titanium (Ti) and its alloys, Fe–Cr–Ni alloys (austenitic SS), magnesium (Mg) alloys, and Co–Cr-based alloys [80]. The advantages and limitations of each of these metals used as implants are presented in Table 1.

### 3.1. Ultra-Fine-Grained Titanium

Ti and its alloys play a major role in the biomedical field because of their high resistance against corrosion in body fluids and excellent biocompatibility. For medical applications, the standard ASTM F-67 has categorized commercially pure Ti (CP Ti) into four grades: G1 to G4. As the technical standard, ASTM F-136 provides the required chemical composition and mechanical properties for Ti grade five (extra low interstitial), which is suitable for the fabrication of surgical implants. Nowadays, dental implants use CP Ti grades four and one as well as an alloy of Ti grade five with a surface treatment for optimizing the contact between the device and the bone, which is known as osseointegration [81]. Nevertheless, Ti of grades two and four is not utilized in medical applications that involve high stresses, such as orthopedic prostheses. For applications such as orthopedic implants, Cr–Co, stainless steel, and grade-five Ti are desirable choices because of their high mechanical resistance. There are some toxic elements, such as V and Al, in most Ti alloys, so commercially pure Ti (CP Ti) has gained more attention. Therefore, V- and Al-free Ti-based alloys have been suggested for biomedical applications [82]. 

SPD methods have been applied to manufacture bulk UFG for medical implants with significantly enhanced mechanical properties and resistance against corrosion [83]. The attachment and proliferation of fibroblasts and osteoblasts are enhanced on nanostructures due to their higher bioactivity, which mimics that of real bone and cause higher adsorption of cell-adhesive proteins [12,84]. The products deformed through SPD have higher wettability and more surface energy [85]. By using SPD techniques, the mechanical properties and wear resistance of Ti alloys are improved by changing the sizes of the grains to the ultra-fine range. In addition, enhanced bioactivity is achieved through an intensified biological response that induces apatite precipitation due to a major increase in cell adhesion/proliferation and higher surface energy [86]. There is a spontaneous layer of titanium oxide that forms on the surface of Ti alloys, making them appropriate for dental and orthopedic implants [87]. This layer protects the surface of metals by making them chemically inert in distinct media. Nevertheless, despite this characteristic, inert Ti is barely able to significantly control any cellular interactions, leading to and sustaining long-term bone synthesis, which is helpful for implants [88]. Much research has been done on the effect of UFG materials on improving the osseointegration between the surfaces of materials and adjacent bone formations due to a similar topography with bone itself [89,90,91,92,93].

One of the earliest studies on cell responses (in vitro) to UFG metals to which SPD was applied was done by Kim et al. [94], who examined the behavior of mouse 3T3 fibroblast cells on UFG CP Ti fabricated using ECAP. Samples made of coarse grain CG Ti and Ti6Al4V alloys were used as controls as well. Contact angle measurements revealed a strong relationship with grain size, showing improved wettability due to the UFG structure. Moreover, there was a considerable increase in the proliferation of cells. In 2004, Webster et al. showed that nano-grained Ti6A14V, Ti, and CoCrMo produced using traditional powder metallurgy enhanced the adhesion of osteoblasts [91]. In another study, Tevlek et al. used a severe shot peening technique to create a gradient layer structure. They found that MC3T3-E1 cell proliferation was enhanced without any changes in surface chemistry [3]. Bindu et al. [95] conducted the first in vivo assay on pure G2 Ti treated using the ECAP technique. They focused on sample biocompatibility and assessment of inflammatory cells—particularly macrophages—around the implant. The fabricated models were implanted into Wistar rats. After 30 days, the implants and their surrounding tissues were analyzed to detect the macrophage numbers. Based on their results, there were fewer macrophages on the fabricated samples compared with the untreated ones. Table 2 summarizes some of the more recent studies using SPD processes, in terms of the materials and the types of cells studied. 

**Figure 11:**
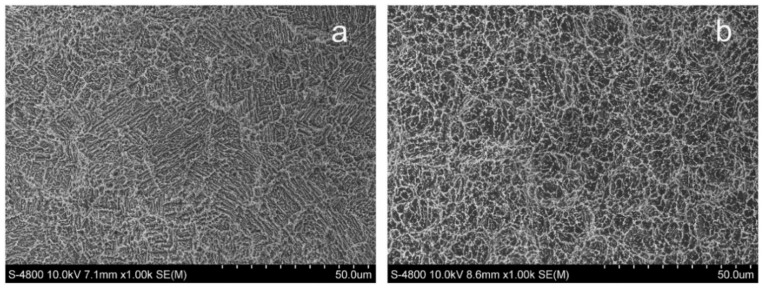
Scanning electron microscopy (SEM) micromorphology of UFG Ti (**a**) and CP Ti (**b**) adapted from [10], with permission from Elsevier, 2020.

An et al. [10] created UFG Ti via ECAP by applying sandblast and acid etching and evaluated the biocompatibility of the UFG Ti as a dental implant. The CP Ti surface (Figure 11a) showed a rough microstructure with large indentations that were dozens of micrometers in diameter under low magnification, while the UFG Ti surface (Figure 11b) presented smaller uniform indentations that were micrometers in diameter. Several hierarchical porous topographies were shaped on the surface of the UFG Ti and CP Ti.

**Figure 12:**
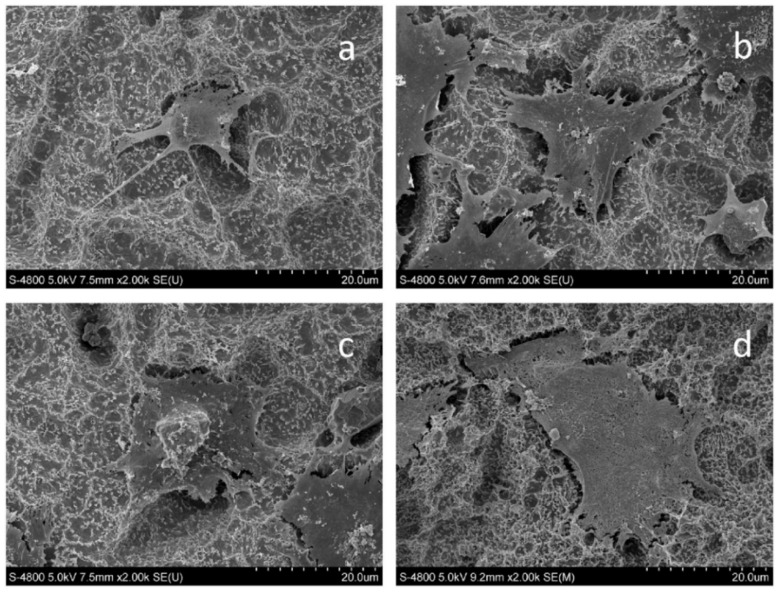
SEM figures of MC3T3-E1 cells on the surfaces of CP Ti (**a**,**b**) and UFG Ti (**c**,**d**). In these images, (**a**,**c**) and (**b**,**d**) show cell adhesion after 2 and 24 h of incubation, respectively, adapted from [10], with permission from Elsevier, 2020.

They seeded MC3T3-E1 osteoblasts onto the samples to investigate their (in vitro) biocompatibility. For the (in vivo) assay, UFG Ti implants were embedded into the rabbit’s femurs, and CP Ti was selected as the control group. Based on their results, the cells cultured on UFG Ti showed more adhesion, proliferation, and viability in comparison with the control group (Figure 12).

**Figure 13:**
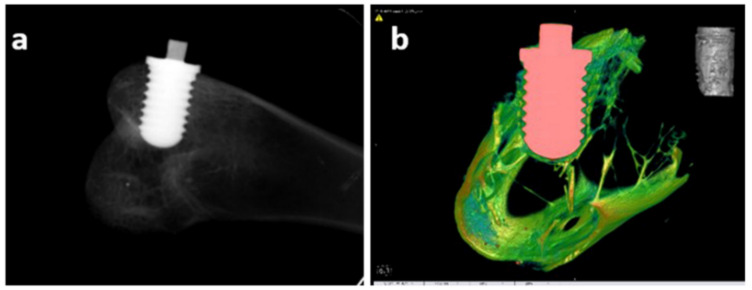
X-ray film (**a**) and reconstructed micro-CT image (**b**) from a model with an implant. The illustration in the upper-right corner of image (**b**) is the reconstructed image of the bone within the region of interest, adapted from [10], with permission from Elsevier, 2020.

Moreover, using an in vivo assay, desirable osseointegration occurred between the implant and bone in both groups, but the intensity of the combination of the UFG Ti with the bone was greater based on pull-out tests. Figure 13a shows an X-ray examination of the bone–implant interface, which showed no bone absorption. Furthermore, a new bone matrix was detected around the implant in the reconstructed micro-CT images (Figure 13b).

### 3.2. Ultra-Fine-Grained Stainless Steel

In comparison with other metallic alloys, 316 L stainless steel (SS) is a suitable candidate for orthopedic wires and screws, cardiovascular applications, artificial joints, and spinal fixation devices because of its suitable biocompatibility, high resistance against corrosion, high mechanical strength, cost effectiveness, and simple production process [108,109,110]. The corrosion of localized pitting is the main disadvantage of SS. In comparison with 316 L SS, AISI 304 austenitic SS showed high resistance against corrosion because of the passive self-repairing oxide film on its surface, which showed better biocompatibility due to its lower nickel ratio. However, deterioration of this layer may happen because of corrosion and the surface of the materials becoming sensitive to corrosion [3]. Many dental and surgical implants are made from face-centered cubic (FCC) crystalline structures of stainless steel at room temperature because of their low risk of thrombosis and notable in vitro and in vivo biocompatibility [111]. Several research groups have published various surface treatment methods that induce grain refinement on stainless steel surfaces (Table 3). Idell et al. [112] obtained a grain refinement of around 42 nm on the surface of 316L SS through SPD induced by machining. In recent research, Yin et al. [113] fabricated extremely fine nano-grained 316L SS with an average grain size of 10 nm via an ultrasonic shot peening technique. Micropillar compression and nanoindentation tests showed a considerable enhancement in yield strength and nano-hardness. Moreover, in vitro results revealed a significant enhancement in human osteoblasts compared with the as-received coarse-grained 316L SS surface. This improvement can be related to the high number of ultra-high-density boundaries with nanosized grains, which could obstruct dislocation movement when experiencing plastic deformation and promote the adsorption of proteins by providing a continuum of probable protein adsorption sites with partial surface coverage when encountering biological environments.

### 3.3. Ultra-Fine-Grained Magnesuim

Magnesium and its alloys are increasingly being used in different industries, such as aerospace and electronics, due to their lightness and excellent mechanical properties. Magnesium-based alloys are promising candidates for orthopedic implants as well, as they can reduce the risk of stress shielding of implants because their mechanical properties are similar to those of bone tissue. At the same time, they are considered resorbable implants, as they dissolve and can be replaced by bone tissue, which clears out the need for surgically removing temporary implants. However, the corrosion rates and the leaching rates of different elements (e.g., zirconium and aluminum) of magnesium alloys need to be carefully controlled to ensure their safety for biomedical applications. While magnesium is considered a biocompatible element that naturally exists in bone tissue, its alloying elements have shown toxicity in the body, and choosing biocompatible alloying elements is a vital factor for biomedical applications. Conventional commercial magnesium alloys (AZ31, AZ91, AE21, LAE442, and WE436) [118,119] have been used in the fabrication of biomaterials, and on top of them, newly designed Mg–Ca-based, Mg–Zn-based, Mg–Si-based, Mg–Sr-based, Mg–RE-based, and Mg–Cu-based alloys have emerged. Nevertheless, AZ alloys usually contain a small amount of aluminum, and WE436 contains zirconium [119].

However, the strength of magnesium alloys and their corrosion rates are often not sufficient for orthopedic applications, and SPD processes have been used for grain refinement and improvement of their mechanical properties. Different techniques (such as ECAP, ARB, and CPG, as well as forging, extrusion compression, shear extrusion, and angular rolling techniques) have been used for the grain refinement of such alloys. For example, Nayak et al. used a hot-rolling process at around 310 °C to reduce the grain size of the MZ3 (Mg-Zn based) alloy (82.62%) to around 30 µm, which drastically increased the toughness, strength, and ductility of the material (~264%) [120]. However, the corrosion rate and ion leaching from the materials increased by 35.06% and resulted in a significant drop in the viability of MG 63 cells (human osteosarcoma cells). On the other hand, Saha et al. showed that reducing the corrosion rate of Mg alloys from 1.64 to 0.11 mm/year by using grain refinement with Zr addition enhanced osteoblast cell activity and proliferation [121]. In another study, Silva et al. compared magnesium alloys processed with hot rolling, ECAP, and HPT for their physical properties and in vitro biological response [122]. Their results demonstrated that HPT decreased the grain size of the CP magnesium more than other techniques, from ~480 to around ~0.56 µm. The HPT-processed material exhibited the highest corrosion resistance based on its polarization curve; however, it demonstrated the highest mass loss rate (for the first three days) and, thus, the worst cell response against osteoblasts within 24 h of incubation with cells. Table 4 presented a summary of study on UFG magnesium produced by SPD. 

## 4. Conclusions and Future Perspectives

In recent years, severe plastic deformation processes have developed into unique techniques for obtaining nano-/ultra-fine-grained structures for various metallic materials. Different SPD techniques, known as metal forming processes, that impose an ultra-large plastic strains on bulk materials in order to fabricate UFG metals were reviewed here. The lifetime functionality and mechanical properties of medical implants in the human body have been improved by these techniques through grain refinement. Such processes provide a possibility of implementing materials that could not originally be utilized in physiological conditions in their coarse-grained state because of their poor mechanical properties. Moreover, by using such techniques, the use of expensive, allergenic, or toxic alloying elements is not required. In addition, these techniques lead to downsizing of medical devices or implants and reduce the risk of surgical intervention. Ti and stainless steel processed by SPD methods showed enhanced surface energy and wettability properties in comparison with untreated ones. Furthermore, improved cell adhesion, proliferation, differentiation, and phenotypic expression have been observed in such nano-grained materials. Although numerous studies have been provided here regarding the grain size effect and cellular interactions, as well as mechanical and biological properties, more organized research is still required in order to delineate the mechanisms of better cell responses to nano-grained materials. In addition, most attempts have focused on the short-term evaluation of cell responses to these materials, and further in vivo studies are needed to confirm their effects on bone responses. To get to the point, SPD techniques have been shown to provide unique and new orthopedic and dental implants with enhanced biocompatibility and mechanical properties. Generally, it can be concluded that these nano-/ultra-fine-grained metallic biomaterials have improved bioactivity, better tissue interactions, and higher bone healing rates. Based on the studies covered here, in the near future, it is clear that nano-/ultra-fine-grained materials created with SPD techniques will provide a significant breakthrough in metallic biomaterials with unique properties for numerous biomedical applications.

## Figures and Tables

**Table 1:** An overview of metals used as biomedical implants.

Metals/Alloys	Benefits	Limitations
**Commercially pure titanium (CP Ti)**	- High biocompatibility - High resistance against corrosion - Direct apposition on bones - Moderately low elastic modulus	- Poor fatigue and static strength for application in load-bearing implants - Low resistance against wear
**Ti–6Al–4V**	- Significant resistance against corrosion - Great biocompatibility - Direct apposition on bones - Moderately low elastic modulus - High strength in fatigue and static tests	- Low wear resistance - Aluminum and vanadium ion release may cause health problems
**Stainless steel 316 L**	- Excellent ductility - High resistance against wear - Good machinability	- Lower fatigue strength compared to other alloys used in implants - High elastic modulus - Poor resistance against corrosion and biocompatibility in comparison with other implants - Relatively high release of metal ions and adverse response of host organs
**Co–Cr-based**	- Excellent strength of static and fatigue - High resistance against corrosion - Good biocompatibility	- Great elastic modulus - Lower corrosion resistant and biocompatibility compared with Ti alloys - Unfavorable host response to released Ni and Cr ions
**NiTi (Nitinol)**	- Good biocompatibility and corrosion resistance - Shape memory and high elastic effects - Poor stiffness	- Adverse response of host organs to released Ni ions - Low wear resistance - Complicated production procedure
**Magnesium-based**	- Magnesium has good biocompatibility - Biodegradability - Lightness and low density - Good toughness	- Rapid corrosion rate - Leaching of magnesium may cause health problems - Innovative fabrication and treatment processes are required for biomedical applications

**Table 2:** Summary of studies on ultra-fine-grained (UFG)/Nano grain NG titanium produced using SPD.

Materials/Alloys	SPD Process	Size of Grains/Instrument	Applications	In Vivo/In Vitro	Significant Findings	Ref.
Titanium Grade 2	Hydrostatic Extrusion (HE)	92 (nm), TEM	Bone tissues	SaOS-2 cells	- Useful effects on SaOS-2 cell proliferation and attachment and protein adsorption. - Increased biocompatibility of titanium after the HE process. - Greater homogeneity of the oxide layer on the surface of Ti and improved resistance against corrosion.	[83]
β-type Ti-13Nb-13Zr (TNZ)	Hydrostatic Extrusion (HE)	20 (nm)	-	-	- Significant grain refinement and high densities of dislocation. - Increase to 50% of strength. - Slightly lower Young’s modulus values than the initial state.	[96]
Ti13Nb13Zr and Ti35Nb7Zr5Ta	High-Pressure Torsion (HPT)	~203 and ~112 (nm), TEM	-	Osteoblastic cells	- Significant reduction in grain sizes, resulting in UFG microstructures.	[86]
Nanostructured Ti (nTi)	Equal-Channel Angular Pressing	-	Orthodontics	-	- Smoother surface structure and trans-granular fracture aspect for nano Ti mini-implants. - More torsion resistance in comparison with Ti-6Al-4 V and CP Ti mini-implants.	[97]
Ti–6Al–7Nb	Equal-Channel Angular Pressing	200 (nm), TEM	Orthopedic implants		- Increased strength and fatigue properties.	[98]
Commercially pure (CP) Ti Grade 2	Cold Hydrostatic Extrusion	<90 (nm), TEM	Surgical osteosynthesis		- Increased strength with moderate ductility and suitable thermal stability. - Improved the quality of the surface and increased grain refinement.	[99]
CP Ti grade 2 and Ti-6A1-4 V	Equal-Channel Angular Pressing	~23 (nm), TEM	Implants	Mouse fibroblast cell line 3T3	- Enhanced strength and improved biocompatibility, including wettability and cell adhesion/proliferation, compared to conventional Ti. - More surface energy and nano-sized grooves.	[100]
Ti–6Al–4V	Equal-Channel Angular Pressing	~170 (nm), TEM	Dental implants	MG63 cells	- Higher MG63 cell proliferation rate compared to control groups.	[101]
CP Ti	Equal-Channel Angular Pressing	183 (nm), TEM	Implants	Fibroblast cells	- Improved both corrosion and biological behavior.	[102]
CP Ti grade 2 and Ti-6A1-4 V	Equal-Channel Angular Pressing	238 (nm), TEM	Implants	Mouse fibroblast cell line 3T3	- Improved both strength and cell–substrate interactions. - Improved biocompatibility, such as lower contact angles and cell adhesion/proliferation.	[94]
CP Ti	Equal-Channel Angular Pressing	200–300 (nm), SEM	Bone–implant osseointegration	New Zealand rabbits/MC3T3-E1 cells	- Significantly improved yield strength and Vickers hardness. - Excellent cell compatibility.	[10]
CP Ti grade 2 and Ti-6A1-4 V	Equal-Channel Angular Pressing	200–300 (nm), SEM	Dental endosseous implants	MC3T3-E1 pre-osteoblast cells	- Improved spreading, attachment, viability, and alkaline phosphatase ALP activity of cells. - Significantly more ALP and mRNA levels of osteopontin and osteocalcin in cells.	[85]
Commercial coarse-grained pure titanium	Equal-Channel Angular Pressing	200 (nm), ESEM	Bone implants	Osteoblast-like cell line MG63	- Enhanced osteoblast-like cell attachment and in vitro proliferation. - Low rate of corrosion in simulated body fluid.	[103]
Bulk nanocrystalline Ti bars (Grade 4)	Equal-Channel Angular Pressing	250 (nm), TEM	Bone implants	Osteoblast cell lines (MG63)/tibia of Beagle dogs	- Stronger interactions and higher cellular functionalization when cells were co-incubated with Ti implants. - Fresh bone around the implants.	[84]
CP Ti	High-Pressure Torsion (HPT)	10–50 (nm), TEM	Bone implants	Mouse pre-osteoblast MC3T3-E1 subclone 14 and fibroblast cell lines from rats	- Improved cell activity and higher degree of surface wettability. - Promoted cellular response and mechanical properties. - Supported pre-osteoblast attachment and spreading over fibroblasts and enhanced the cytoskeleton and activity of the extracellular matrix.	[90]
CP Ti	Ultrasonic Shot Peening	14–20 (nm), SEM	Dental and orthopedic implants	Human osteoblast cell line, MG 63	- Significant improvement in the proliferation of cells. - Increased resistance against corrosion. - Much more prominently nanostructured surface with promoted density and sharper grain boundaries.	[104]
CP Ti	High-Pressure Torsion (HPT)	10–50 (nm), TEM	Bone implants	Mouse pre-osteoblast MC3T3-E1	- Significantly more attached pre-osteoblast cells and growth rate on the surface of Ti materials.	[92]
CP Ti	Ultrasonic Shot Peening	57–88 (nm), XRD	Bone implants	MG63 cells/new Zealand White rabbits	- Improved cell behavior in (in vitro) assays in comparison with a coarse-grained Ti surface.	[89]
CP Ti grade 2	Equal-Channel Angular Pressing	-	Implants	Murine fibroblast cells 3T3/Wistar rats	- More cytocompatible than untreated samples.	[95]
Ti-Nb-Mo-Zr	Cold Rolling	-	Orthopedic implants	-	- Increased ratio of hardness/Young’s modulus continuously with deformation degree.	[105]
Ti–15Zr	Cold Rolling	2–5 (µm), SEM	Dental implants	-	- Enhanced fatigue performance of Ti–15Zr over Ti–Grade 4. - Higher strength of Ti–15Zr alloy than that of Grade 4 titanium.	[106]
Ti-32.5Nb-6.8Zr-2.7Sn	Cold Rolling	200–250 (nm), OM	Bone implants	-	- The fabricated alloy showed moderate strength, suitable elongation, low elastic modulus, and high elastic admissible strain.	[107]

**Table 3:** Summary of studies on UFG/NG stainless steel as produced by SPD.

Materials/Alloys	SPD Process	Size of Grains/Instrument	Applications	In Vivo/In Vitro	Significant Findings	Ref.
AISI 304 austenitic SS	Severe shot peening (SSP)	Under 300 (nm), FESEM	Implants	MC3T3-E1 pre-osteoblast	- Promoted metal surface mechanical properties. - Enhanced cellular behavior - and increased proliferation of cells.	[3]
316L SS	Severe shot peening	25 (nm), XRD	Orthopedic implants	Osteoblasts	- Noticeable improvement in work hardening of the surface and a residual of compressive stresses. - Kept the adhesion and proliferation of osteoblasts. - Significant decrease in the growth of S. aureus and S. epidermidis adhesion.	[114]
316L SS	Ultrasonic Shot Peening	10 (nm), FIB channeling contrast imaging technique	Orthopedic implants	Human osteoblast cells (SaoS-2)	- Significant improvement in human osteoblast cell (Saos-2) attachment. - Noticeable enhancement of nanohardness and yield strength of the developed NG-316L SS.	[113]
316L SS Sheet	Ultrasonic shot peening	Less than 50 (nm), SEM	Orthopedic implants	MC3T3-E1 subclone 4	- Enhanced osteoblast attachment and proliferation.	[115]
316L SS	Ultrasonic shot peening	326 (nm), SEM	Orthopedic implants	Human osteoblast cells	- Significant improvement in the behavior of human osteoblasts.	[116]
316L SS	Equal channel angular pressing	78 (nm), SEM	Implant	Fibroblast cells	- Significant decrease in corrosion rate. - Dramatic enhancement in cell proliferation.	[108]
316L SS	Severe shot peening	100–200 (nm), SEM	Bone implants	Human osteoblasts	- Induced compressive residual stresses and work hardening in the top layer of the surface. - Promoted osteoblast cell spreading and enhanced expression of focal adhesion proteins.	[117]

**Table 4:** Summary of studies on UFG/NG magnesium as produced by SPD.

Materials/Alloys	SPD Process	Size of Grains/Instrument	Applications	In Vivo/In Vitro	Significant Findings	Ref.
WE43 (Mg-Y-Nd-Zr)	ECAP	0.73 (µm), TEM	Implants	Red blood cells and white blood cells	- Improved mechanical properties. - No change in electrochemical corrosion. - Enhanced cellular response and biocompatibility.	[123]
